# Bioengineered Anti-PD-L1 Functionalized Nanoplatform for Targeted Delivery and Tumor Immune Reprogramming Against Colorectal Cancer

**DOI:** 10.34133/bmr.0284

**Published:** 2025-12-12

**Authors:** Miao Liu, Xinjuan Ma, Ruijie Zhou, Xiaojuan Yang, Yongsheng Zhou, Bin Ma, Chunxia Su, Xiangguo Duan

**Affiliations:** ^1^ School of Inspection, Ningxia Medical University, Yinchuan 750004, China.; ^2^ The First School of Clinical Medicine, Ningxia Medical University, Yinchuan 750004, China.; ^3^ The Second School of Clinical Medicine, Ningxia Medical University, Yinchuan 750004, China.; ^4^ Department of Oncology Surgery, The First People’s Hospital of Yinchuan, Yinchuan 750004, China.; ^5^ School of Basic Medical Sciences, Ningxia Medical University, Yinchuan 750004, China.

## Abstract

Colorectal cancer (CRC) remains a major clinical challenge owing to its immunosuppressive tumor microenvironment and limited targeting therapeutic efficiency. Developing innovative strategies that integrate immune activation with enhanced tumor-targeting ability is urgently needed. Herein, we reported a bioengineered exosome drug delivery nanoplatform (Apatinib-Exo^aPD-L1^), in which HEK293T-derived exosomes were surface functionalized with anti-PD-L1 antibody (aPD-L1) and encapsulated the tyrosine kinase inhibitor Apatinib, aiming to enhance the tumor-targeted immunotherapy against CRC. Apatinib-Exo^aPD-L1^ exhibited efficient tumor-targeting capability and prolonged systemic circulation, attributed to aPD-L1 modification, resulting in markedly enhanced antitumor efficacy without evident body toxicity. Mechanistically, Apatinib was efficiently delivered and internalized by tumor cells, where it triggered immunogenic cell death (ICD) and promoted dendritic cell maturation. This immune activation cascade facilitated the infiltration and activation of cytotoxic T cells within the tumor microenvironment. Furthermore, Apatinib-Exo^aPD-L1^ reduced the population and suppressive function of regulatory T cells (Tregs) and myeloid-derived suppressor cells (MDSCs), thereby effectively reversing immune suppression and amplifying the antitumor immune response. Collectively, our findings demonstrated that Apatinib-Exo^aPD-L1^ is a safe and effective exosome-based therapeutic platform, offering a promising strategy to convert immunologically “cold” tumors into “hot” ones and improve clinical outcomes in CRC.

## Introduction

Colorectal cancer (CRC) is one of the most prevalent malignant cancers worldwide, ranking third in incidence and second in mortality among all malignancies, and it poses a major public health challenge [1]. Multiple factors, including microsatellite instability, inflammation, and microRNAs, contribute to CRC pathogenesis [2]. Although advancements in surgical resection, radiotherapy, and chemotherapy have improved patients’ outcomes, their efficacy remains limited due to substantial side effects and development of drug resistance, both of which contributed to therapeutic failure. Notably, nearly 30% to 40% of patients experience recurrence and metastasis after adjuvant therapy, emphasizing the urgent need for innovative therapeutic strategies [3,4].

Exosomes, with diameters ranging from 30 to 200 nm, have emerged as a promising alternative to synthetic nanoparticles in cancer therapy [5]. Exosomes originate from multivesicular bodies and facilitate intercellular communication by transferring bioactive molecules such as lipids, proteins, and nucleic acids. The biologically active substances depend on their cellular origin and confer distinct functions of exosomes [6,7]. Recent studies showed that natural killer cell-derived exosomes exert potent antitumor effect by activating the FAS/FASL pathway through their strong cytotoxic activity. [8]. Tumor cell-derived exosomes are able to promote the formation of the tumor microenvironment (TME) and promote the migration of tumor cells. Importantly, exosomes have emerged as highly promising drug delivery vehicles owing to their high biocompatibility, low immunogenicity, inherent targeting ability, and minimal toxicity [9,10]. To address nonspecific biodistribution, engineered exosomes, especially those with surface modifications including targeting ligands or antibody decorations, have shown improved specificity, improved drug pharmacokinetics, and reduced systemic toxicity in delivering chemotherapeutic agents, immune modulators, and inhibitors, thereby enhancing cellular uptake and in vivo accumulation compared with unmodified vesicles [11,12]. For instance, exosomes engineered to display anti-PD-L1 (aPD-L1) via Fc-binding modules markedly enhanced cellular uptake efficiency in HER2^+^ breast cancer and melanoma models, and further improved in vivo antitumor efficacy when co-delivered with doxorubicin (DOX) [12]. Beyond improving biodistribution, exosomes are also being increasingly combined with immunotherapeutic strategies to modulate the tumor immune microenvironment. Exosomes decorated with anti-CD47 antibody for the co-delivery of cisplatin not only blocked the CD47-mediated “don’t eat me” signal and promoted macrophage-mediated phagocytosis of tumor cells, but also markedly enhanced the antitumor efficacy of cisplatin to overcome chemoresistance in non-small cell lung cancer (NSCLC) [13]. Notably, dual checkpoint blockade was achieved by co-delivering PD-L1 and CTLA-4 small interfering RNAs (siRNAs) via exosomes, thereby repressing malignant features of CRC cells, restraining tumor growth, and activating tumor immune responses [14]. Furthermore, delivery of STING agonists via engineered exosomes amplified type I interferon responses and synergized with checkpoint blockade, resulting in enhanced dendritic cell (DC) activation and cytotoxic T lymphocyte (CTL) infiltration while reducing systemic toxicity [15]. These advances suggest that antibody-functionalized exosomes may serve as an effective platform for enhancing both therapeutic targeting and immune modulation in cancer treatment.

Immunotherapy, particularly immune checkpoint inhibitors (ICIs) such as aPD-1/PD-L1 and aCTLA-4 antibodies, has revolutionized cancer treatment in recent years [16,17]. However, their clinical efficacy in CRC, especially in recurrence or advanced cases, remains limited owing to the intrinsically low response rates [18]. The PD-1/PD-L1 axis plays a key role in tumor immune evasion by suppressing T cell activation and promoting immune tolerance, thereby contributing to the establishment of an immunosuppressive TME, making PD-L1 as a crucial target in cancer immunotherapy. Mechanistically, studies show that the PD-1/PD-L1 interaction inhibits the phosphatidylinositol 3-kinase/protein kinase B/mammalian target of rapamycin (PI3K/AKT/mTOR) signaling pathway, thereby leading to PTEN upregulation, while concurrently reducing the infiltration of pro-inflammation T helper 17 (Th17) [19] and Th1 [20] cells, as well as enhancing the activation of regulatory T cells (Tregs). These changes contribute to the formation of an immunosuppressive TME and are considered as key factors underlying the poor responsiveness of immunologically “cold” tumors to ICI-based therapies [21,22].

Recent studies have highlighted the importance of reprogramming the TME to improve immunotherapeutic outcomes [23]. One promising strategy involves the induction of immunogenic cell death (ICD), which acts as a bridge between tumor cell death and immune activation. A variety of therapeutic interventions, including photodynamic therapy, hyperthermia, tyrosine kinase inhibitors (TKIs), and chemotherapeutic agents such as DOX and oxaliplatin, have been shown to induce ICD through the release of damage-associated molecular patterns (DAMPs), tumor-associated antigens (TAAs), and pro-inflammatory cytokines [24–26]. Hallmarkers of ICD molecules such as adenosine triphosphate (ATP), high mobility group box 1 (HMGB1), calreticulin (CRT), and heat shock proteins (HSPs), engage phagocytic receptors on DCs, leading to antigen processing and presentation via major histocompatibility complex class I (MHC-I). This cascade facilitates CTL activation and infiltration and is accompanied by increased cytokine secretion such as interferon-γ (IFN-γ) and tumor necrosis factor-α (TNF-α), which subsequently initiate robust adaptive immune responses and drive the conversion of “cold tumors” into “hot” ones [23,25,26].

Apatinib, a selective small-molecule TKI targeting vascular endothelial growth factor receptor-2 (VEGFR-2), has demonstrated broad antitumor activity in multiple malignancies, including CRC. Beyond its potent antiangiogenic effects, Apatinib has been shown to modulate tumor-associated signaling pathways such as PI3K/AKT/mTOR and ROS/Nrf2/p62, thereby promoting tumor cell apoptosis and enhancing sensitivity to chemotherapeutic agents [27,28]. Moreover, its synergistic effect with immunotherapies highlights its potential to reshape the tumor immune microenvironment and improve the therapeutic outcomes. However, despite these promising therapeutic effects, the clinical application of Apatinib remains limited owing to several drawbacks such as poor water solubility, nonspecific tissue distribution, and dose-dependent systemic toxicity. Multiple clinical studies have reported that Apatinib may induce adverse events, including hypertension, proteinuria, and liver injury, which markedly affect patient compliance [29,30]. Nevertheless, despite recent advances in exosome engineering and immune checkpoint blockade therapy, few studies have systematically integrated antibody-functionalized exosomes with small-molecule TKIs to simultaneously enhance tumor targeting and immunomodulation in CRC, and their specific mechanisms have not been elucidated.

In this study, we developed a bioengineered exosome platform by conjugating aPD-L1 onto HEK293T-derived exosomes with Apatinib loading to improve targeted delivery and enhance immunotherapy against CRC. Specifically, aPD-L1 was anchored onto the exosomal surface to facilitate tumor-specific targeting, while Apatinib was encapsulated within the vesicles to induce ICD and stimulate immune activation. Compared with unmodified systems, this dual-functional nanoplatform exhibited enhanced tumor accumulation, prolonged circulation time, efficient intracellular delivery, and robust induction of antitumor immune responses. These findings highlight the therapeutic potential and biosafety of Apatinib-Exo^aPD-L1^ for remodeling the immunosuppressive TME and converting immunologically “cold” tumors into “hot” ones, ultimately improving clinical outcomes.

## Materials and Methods

### Antibody-conjugated peptide synthesis

1,2-Distearoyl-sn-glycero-3-phosphoethanolamine-polyethylene glycol-N-hydroxysuccinimide (DSPE-PEG-NHS) (R-0042-2k, Ruixibio, China) was dissolved in dimethyl sulfoxide (DMSO) and mixed with aPD-L1 at a molar ratio of 1:2. The mixture was continuously stirred at 25 °C for 24 h. Afterward, the mixture was placed in a dialysis bag and dialyzed for 48 h to remove un-conjunctive DSPE-PEG-NHS linkers.

### Preparation of Apatinib-Exo^aPD-L1^

HEK293T cells were cultured until 80% confluence, and the medium was then replaced with culture medium containing exosome-free serum incubated for 48 h. Exosomes were purified by a differential centrifugation method as described above [31]. The culture medium was collected and centrifuged at 2,000*g* at 4 °C for 10 min to remove the cells, followed by centrifuging at 10,000*g* at 4 °C for 30 min to remove the debris and apoptotic body, and then the supernatant was further centrifuged at 100,000*g* for 70 min using an ultracentrifuge (Beckman Coulter, Brea, USA). The pellet was resuspended in phosphate-buffered saline (PBS) and ultracentrifuged for another 70 min at 100,000*g* at 4 °C. The pellet containing exosomes was resuspended in 100 μl of PBS. The protein concentrations of the exosomes were measured using the bicinchoninic acid (BCA) assay. Then, Apatinib was dissolved in DMSO and co-incubated with isolated exosomes via sonication to facilitate drug loading into the exosomes. The drug loading capacity (DLC) and encapsulate efficiency (EE) of Apatinib were quantitated by ultraviolet spectrophotometry (UV–Vis). The DSPE-PEG-aPD-L1-conjugated peptide was co-incubated with the isolated exosomes. The mixture was ultrafiltered at 4,500*g* for 10 min to remove free DSPE-PEG-aPD-L1, and the antibody conjugated efficiency was quantified by BCA and fluorescence.

### Characterization of Apatinib-Exo^aPD-L1^

Malvern Mastersizer was used to measure the size and zeta potential of the exosomes (NanoSight, Malvern, UK). The structure and morphology of exosomes were visualized with a transmission electron microscope (TEM). For Western blot analysis, exosome lysates were subjected to electrophoresis using 4% to 20% polyacrylamide gels and transferred onto polyvinylidene difluoride (PVDF) membranes (Amersham, Cytiva, USA). The membranes were blocked with 5% milk at room temperature for 1.5 h. The membranes were incubated with Alix (PDCD6IP, Abways, 1:1,500 dilution), TSG101 (WL05130, Wanleibio, 1:1,000 dilution), CD9 (ab236630, Abcam, 1:1,000 dilution), and calnexin (66903-1-Ig, Proteintech, 1:10,000 dilution) primary antibodies at 4 °C overnight followed by incubation with horseradish peroxidase (HRP)-conjugated goat anti-rabbit immunoglobulin G (IgG) (66031-1-Ig, Proteintech,1:5,000 dilution) secondary antibody for 1.5 h at room temperature. Protein bands were visualized using an Amersham Imager 600 system (Cytiva, USA).

### Hemolysis test

Blood samples were obtained from BALB/c mice. Erythrocytes were isolated by centrifugation at 1,500 rpm for 20 min and washed 3 times with PBS. Erythrocyte suspension (2%) was prepared by diluting the erythrocyte with 0.9% NaCl. Next, Apatinib-Exo^aPD-L1^ at various concentrations (15, 30, 60, 120, 240, and 300 μg/ml) was added to 2% erythrocyte suspension at the ratio of 1:1 and incubated at 37 °C for 3 h. Afterward, the mixture suspensions were centrifugated at 12,000 rpm for 20 min and photographed. Water served as the positive control, and 0.9% NaCl was applied for the negative control.

### Internalization of exosomes

To examine the internalization of exosomes, SW620 cells were seeded into 12-well chamber slides in Dulbecco’s modified Eagle’s medium (DMEM) supplemented with 10% exosome-free fetal bovine serum (FBS) (VivaCell Biosciences, China) for 24 h and incubated with the Dio-labeled exosomes at 37 °C for 8 h. The cells were then fixed with 4% paraformaldehyde for 15 min, followed by staining with 4′,6-diamidino-2- phenylindole (DAPI) for nuclear staining. The internalization efficiency was observed with a fluorescence microscope (Nikon, Japan).

### Cell apoptosis by flow cytometry (FCM)

To validate cell apoptosis rates, briefly, SW620 and CT26 were seeded in 12-well plates at a cell density of 3 × 10^5^ per well and incubated for 24 h. Afterward, cells were harvested after 48h treatment with PBS, Exo, aPD-L1, Exo^aPD-L1^, Apatinib, Apatinib-Exo, and Apatinib-Exo^aPD-L1^. Collected cell pellets were washed twice with PBS, and cells were stained with Annexin V–fluorescein isothiocyanate (FITC) and propidium iodide (PI) for 15 min. The apoptotic cells were examined with a flow cytometer (FACSCelesta, BD, USA) within 1 h.

### Live/dead cell staining assay

SW620 and CT26 were seeded in 12-well plates at a cell density of 1 × 10^5^ per well and incubated for 24 h. Afterward, the cells were treated with PBS, Exo, aPD-L1, Exo^aPD-L1^, Apatinib, Apatinib-Exo, and Apatinib-Exo ^aPD-L1^. Then, calcein-AM and pyridinium iodide (PI) were used to stain the live cells (green) and the dead cells (red). The apoptosis rates of the cells were observed with a fluorescence microscope (Nikon, Japan).

### Wound healing assay

SW620 and CT26 were seeded in 6-well plates at a cell density of 5 × 10^5^ per well and incubated for 24 h. Then, the cells were scratched and washed with PBS. Cell culture medium was replaced with serum-free DMEM containing PBS, Exo, aPD-L1, Exo^aPD-L1^, Apatinib, Apatinib-Exo, and Apatinib-Exo ^aPD-L1^ for 48 h. The images of the scratch areas were captured at 0 and 48 h with an inverted microscope (DM3000, Leica, Germany), and cell migration areas were quantified by ImageJ (NIH, USA).

### CRT expression and HMGB1 detection

SW620 and CT26 cells were seeded in 12-well plates at a cell density of 1 × 10^5^ per well and incubated for 24 h. The cell culture medium was refilled with PBS, Exo, aPD-L1, Exo^aPD-L1^, Apatinib, Apatinib-Exo, and Apatinib-Exo^aPD-L1^ for 48 h. Afterward, the cells were fixed with 4% paraformaldehyde for 20 min and then incubated with anti-CRT (27298-1-AP, Proteintech, 1:200 dilution) primary antibody to detect CRT exposure. For CRT and HMGB1 detection by Western blot, SW620 and CT26 cells were lysed with radioimmunoprecipitation assay (RIPA) lysis buffer. Equal amounts of proteins were separated by sodium dodecyl sulfate–polyacrylamide gel electrophoresis (SDS-PAGE) and transferred to 0.45-μm polyvinylidene fluoride (PVDF) membranes (Amersham, Cytiva, USA). After blocking with 5% milk for 1.5 h at room temperature, the membranes were incubated overnight at 4 °C with anti-CRT (10292-1-AP, Proteintech, 1:1,000 dilution), anti-HMGB1 (10829-1-AP, Proteintech, 1:20,000 dilution), and anti-glyceraldehyde-3-phosphate dehydrogenase (GAPDH) (10494-1-AP, Proteintech, 1:20,000 dilution) primary antibodies. Following 3 washes with phosphate-buffered saline with Tween-20 (PBST), the PVDF membranes were incubated with HRP-conjugated goat anti-rabbit IgG (66031-1-Ig, Proteintech, 1:5,000 dilution) and HRP-conjugated goat anti-mouse IgG (68847-1-Ig, Proteintech, 1:5,000 dilution) secondary antibodies, and protein bands were visualized using an Amersham Imager 600 system (Cytiva, USA).

### BMDC maturation study in vitro

Bone marrow-derived dendritic cells (BMDCs) were isolated from BALB/c mice. RMPI 1640 culture medium supplemented with 10% FBS, granulocyte-macrophage colony-stimulating factor (GM-CSF) (20 ng/ml), and IL-4 (10 ng/ml) was used to induce BMDC differentiation. For in vitro BMDC maturation study, SW620 and CT26 were seeded into the upper chamber of transwell and cultured overnight, followed by the treatment with PBS, Exo, aPD-L1, Exo^aPD-L1^, Apatinib, Apatinib-Exo, and Apatinib-Exo^aPD-L1^. BMDCs were then harvested and seeded to the lower chamber of transwell and cocultured for another 48 h. Finally, BMDCs were collected and stained by anti-CD11c, anti-CD80, and anti-CD86, and the maturation of DC was analyzed via flow cytometry (FACSCelesta, BD, USA).

### Tumor xenograft model establishment

Four-week-old BALB/c mice were purchased from SPF Biotechnology. Mice were maintained in accordance with the guidelines of the Institutional Animal Care and Use Committee of Ningxia Medical University. Approximately 2 × 10^6^ CT26 cells were suspended in PBS and subcutaneously injected into the left armpits of BALB/c mice. When the tumor volumes reached about 200 mm^3^, mice were randomly divided into PBS, Exo, aPD-L1, Exo^aPD-L1^, Apatinib, Apatinib-Exo, and Apatinib-Exo^aPD-L1^ group (*n* = 5). Specifically, mice were randomly assigned to receive one of the following treatments via intraperitoneal injection every 3 d for a total of 12 d (on days 0, 3, 6, and 9). In detail, the treatment regimens included PBS (100 μl), Exo (100 μg/100 μl), aPD-L1, and Exo^aPD-L1^ (200 μg/100 μl, calculated based on aPD-L1 concentration). For the treatment groups containing Apatinib, Apatinib-Exo and Apatinib-Exo^aPD-L1^ were administered at a dose of 30 mg/kg/100 μl (Apatinib concentration). Mice were weighed and tumors were measured every 3 d. Tumor volumes were measured and calculated using the following formula: 1/2 *L* × *W*^2^. Once the tumor volumes of the negative control (NC) group reached about 1,500 mm^3^, all mice were sacrificed and the main organs and tumor tissues were taken for hematoxylin and eosin (H&E) staining, immunohistochemical (IHC) analysis, and terminal deoxynucleotidyl transferase–mediated deoxyuridine triphosphate nick end labeling (TUNEL) assay.

### Biodistribution in vivo

1,1'-Dioctadecyl-3,3,3',3'-Tetramethylindotricarbocyanine Iodide (DiR) (100 μM) was used to label Exo, Exo^aPD-L1^, and Apatinib-Exo^aPD-L1^. One hundred microliters of DiR-labeled Exo, Exo^aPD-L1^, and Apatinib-Exo^aPD-L1^ was intraperitoneally injected into CT26 tumor-bearing mice and imaged at a series of time points using IVIS Lumina System for ex vivo imaging, and mice were sacrificed after injection. Tumors and main organs were collected for imaging.

### Analysis of immune cells

Mechanical grinding was used to prepare single-cell suspensions from the spleen, lymph nodes, and tumors of CT26 tumor-bearing mice on day 19 for flow cytometry analysis. Briefly, spleens, lymph nodes, and tumors were collected and ground through a 75-μm cell strainer (YA0961-1EA, Solarbio, China). The cell suspension was carefully added on the top of mouse lymphocyte separation medium (P8860, Solarbio, China) and centrifugated at 2,000 rpm for 15 min at 4 °C. Lymphocytes were collected and treated with red blood cell lysis buffer to lyse red blood cells for 5 min. Then, cells were washed with PBS and harvested by centrifugation at 1,500 rpm for 5 min at 4 °C. To investigate the proportions of immune cells, the cell suspensions from spleen, lymph nodes, and tumor of various groups were stained with anti-CD8, anti-TNF-α, anti-IFN-γ, anti-CD11c, anti-CD86, anti-CD80, anti-CD4, anti-CD25, anti-Foxp3, anti-CD11b, anti-F4/80, anti-CD206, and anti-Gr-1 in the dark, followed by flow cytometry analysis (FACSCelesta, BD, USA).

### In vivo toxicity

BALB/c mice were randomly assigned into 7 groups. Different formulations were injected, and mice were weighed every 3 d. On day 19, mice were sacrificed, and the blood was collected for blood chemistry analysis. In addition, main organs such as liver, heart, spleen, lung, and kidney were removed and washed with PBS, fixed in 4% formaldehyde, and embedded in paraffin for H&E staining.

### Statistical analysis

All statistical analysis was performed using GraphPad Prism 8.0. Quantitative data were presented as mean ± standard deviation (SD). For comparisons involving more than 2 groups, 1-way or 2-way analysis of variance (ANOVA) followed by Tukey’s post hoc test was used to perform multiple comparisons and determine statistical significance between groups. Differences were considered statistically significant at *P* < 0.05.

## Results and Discussion

### Synthesis and characterization of Apatinib-Exo^aPD-L1^

**Fig. 1.**
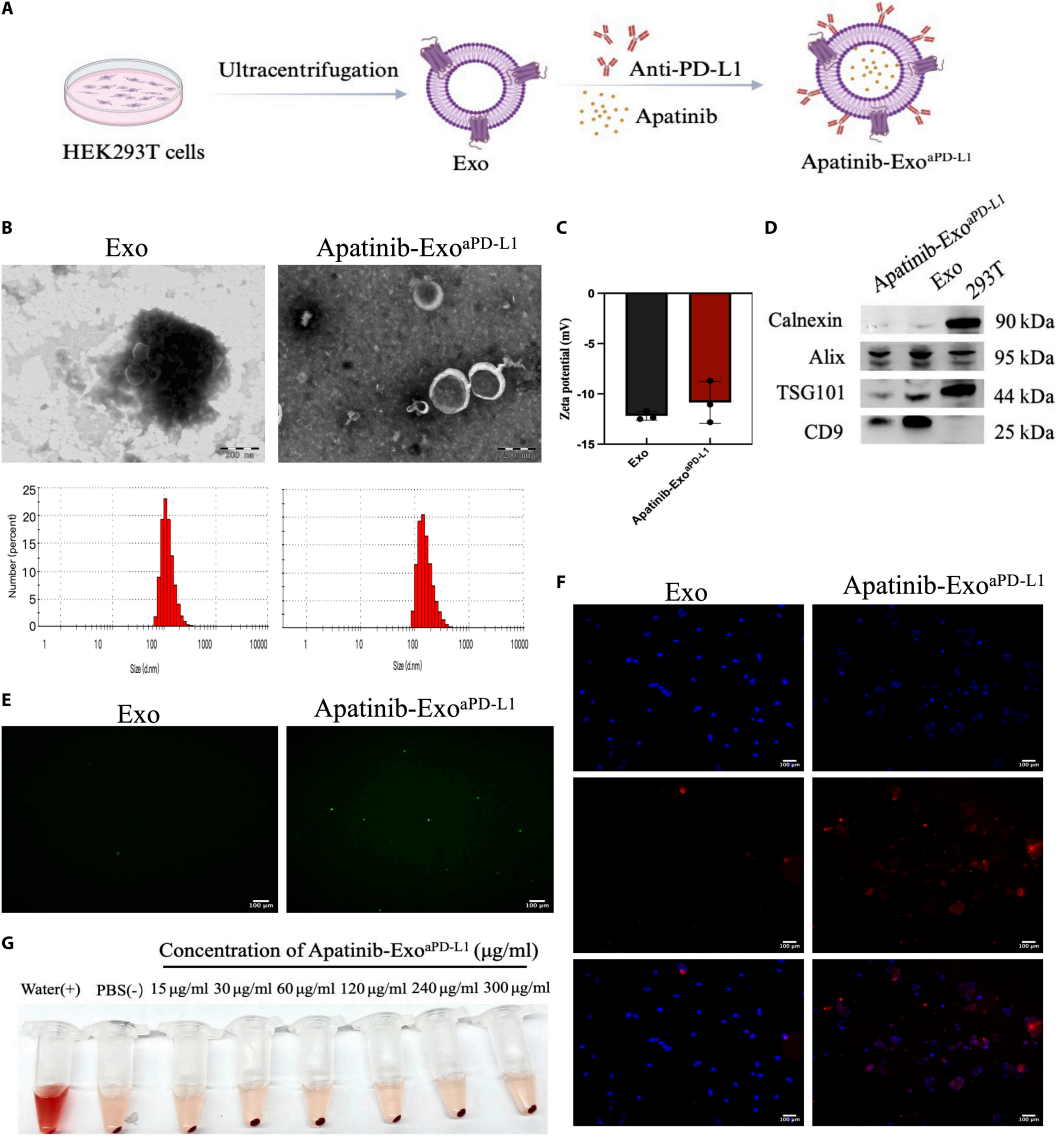
Synthesis and characterization of Apatinib-Exo^aPD-L1^. (A) Schematic illustration of Apatinib-Exo^aPD-L1^ preparation. (B) Representative TEM image and size distribution of Exo and Apatinib-Exo^aPD-L1^. (C) Zeta potential of Exo and Apatinib-Exo^aPD-L1^. (D) Protein expression of the specific biomarkers of exosome analyzed by Western blot. (E) Modification efficiency of aPD-L1 detected by fluorescence. (F) Cellular uptake efficiency of Apatinib-Exo^aPD-L1^ visualized by fluorescence microscope. (G) Representative photographs of 2% erythrocyte suspensions incubated with different concentrations of Apatinib-Exo^aPD-L1^.

HEK293T cells were used as donor cells for exosome production, and Apatinib-Exo^aPD-L1^ was fabricated as the preparation route depicted in Fig. 1A. The morphology and size distribution of Exo and Apatinib-Exo^aPD-L1^ were examined by TEM and analyzed through Malvern Mastersizer. TEM images revealed that the isolated exosomes displayed characteristic spherical shapes with a lipid layer. Similarly, Apatinib-Exo^aPD-L1^ also retained the spherical shapes with Exo, indicating that sonication and antibody modification did not alter the morphology of the naive exosomes. The hydrodynamic diameters of Exo and Apatinib-Exo^aPD-L1^ were found to be approximately 120 nm and 162.7 nm, respectively (Fig. 1B). In addition, the surface charge shifted from −12.18 mV to −10.85 mV after aPD-L1 was conjugated onto the exosome surface, indicating successful surface functionalization of the exosomal membrane and increased stability of the exosomes (Fig. 1C). The observed increases in both particle size and zeta potential following Apatinib loading and aPD-L1 conjugation remained within the typical physicochemical range of exosomes, further supporting the successful incorporation of Apatinib and surface modification with aPD-L1. Meanwhile, Western blot validated the identity of Exo and Apatinib-Exo ^aPD-L1^, as shown in Fig. 1D. Specific protein markers such as TSG101, Alix, and CD9 were presented in both naive and modified exosomes, while the absence of calnexin confirmed the purity of isolated exosomes. Importantly, DLC and EE were quantitated by UV–Vis, which were calculated to be approximately 41.48% and 87.93%, respectively, indicating efficient drug encapsulation within the exosomal vesicles (Fig. S1). Meanwhile, the aPD-L1 conjugation efficiency was evaluated by BCA and fluorescence, showing that when the ratio of aPD-L1 to Exo reached 1:2, the conjugation efficiency reached approximately 97.2%, indicating highly efficient and reproducible surface modification with targeting ligands, which enhanced the specificity of Apatinib-Exo^aPD-L1^ for PD-L1-positive CRC cells (Fig. 1E and Table S1). Next, the tumor-targeting ability of Apatinib-Exo^aPD-L1^ was further evaluated by fluorescence microscope. As shown in Fig. 1F, SW620 cells incubated with Apatinib-Exo^aPD-L1^ exhibited stronger fluorescence signals compared to those treated with naive exosomes, implying that aPD-L1 surface modification markedly improved exosome-mediated cellular uptake. In addition, the hemolysis test was performed to assess the biosafety of Apatinib-Exo^aPD-L1^. The hemolysis rate of Apatinib-Exo^aPD-L1^ remained negligible even at concentrations up to 300 μg/ml, highlighting the biocompatibility of the engineered nanoplatform for systemic administration (Fig. 1G). Together, these results confirm the successful construction of Apatinib-Exo^aPD-L1^ and highlight its characteristics, including appropriate particle size, enhanced tumor-targeting ability, and excellent biocompatibility. These advantages collectively support its potential for efficient delivery and synergistic therapeutic efficacy in CRC.

### Antitumor efficiency of Apatinib-Exo^aPD-L1^ in vitro

**Fig. 2.**
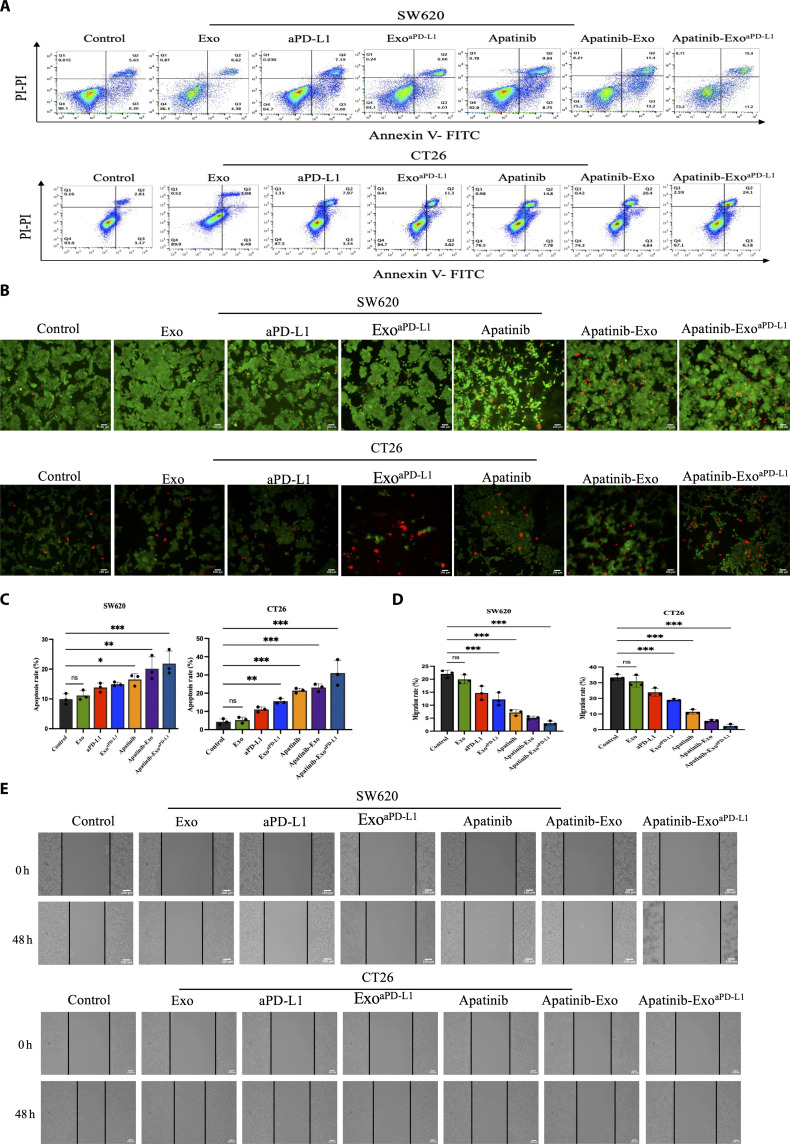
Antitumor efficiency of Apatinib-Exo^aPD-L1^ in vitro. (A) Evaluation of SW620 and CT26 cell apoptosis after different treatments by Annexin V-FITC/PI staining. (B) Fluorescence images of SW620 and CT26 cells stained with calcein-AM (live) and PI (dead) after different treatments. (C) Quantitative analysis of apoptosis rates. (D) Quantitative analysis of migration cells. (E) Microscopy images of SW620 and CT26 cell migration ability after different treatments by wounding healing assay.

To evaluate the antitumor efficiency of Apatinib-Exo^aPD-L1^, we studied its therapeutic performance in vitro. First, to evaluate the apoptotic effects of Apatinib-Exo^aPD-L1^, flow cytometry analysis was performed using Annexin V-FITC/PI staining. SW620 and CT26 cells were treated with PBS, Exo, Apatinib, aPD-L1, Apatinib-Exo, Exo^aPD-L1^, and Apatinib-Exo^aPD-L1^ for 48 h. Results demonstrated that Apatinib-Exo^aPD-L1^ induced significantly higher apoptosis rates compared to the other treatment groups (Fig. 2A). Quantitative analysis revealed that the total apoptotic rate in the Apatinib-Exo^aPD-L1^ group reached 26.6% and 30.28% in SW620 and CT26 cells, respectively, which were markedly higher than those in the Apatinib group (17.79% and 22.58%) and the aPD-L1 group (15.27% and 11.13%). Notably, encapsulating Apatinib into Exo significantly enhanced its apoptotic effect compared with Apatinib or aPD-L1 alone, confirming the potent therapeutic effect of this combinational strategy (Fig. 2C). Consistent results were also observed in calcein-AM/PI live-dead costaining assay. Cells treated with Apatinib-Exo^aPD-L1^ exhibited prominent red fluorescence, indicating extensive cell death and stronger cytotoxicity against CRC cells compared to other groups (Fig. 2B). Additionally, a wound healing assay was performed to evaluate the migration ability of SW620 and CT26 cells treated with different formulations. As shown in Fig. 2D and E, Apatinib-Exo^aPD-L1^ markedly inhibited migration ability of SW620 and CT26 cells, further demonstrating the antitumor activity of Apatinib-Exo^aPD-L1^. Collectively, these findings highlight the enhanced antitumor effect of Apatinib-Exo^aPD-L1^ in vitro, confirming its therapeutic potential as a synergistic nanoplatform for CRC.

### Apatinib-Exo^aPD-L1^ induces ICD effect and promotes DC maturation in vitro

**Fig. 3.**
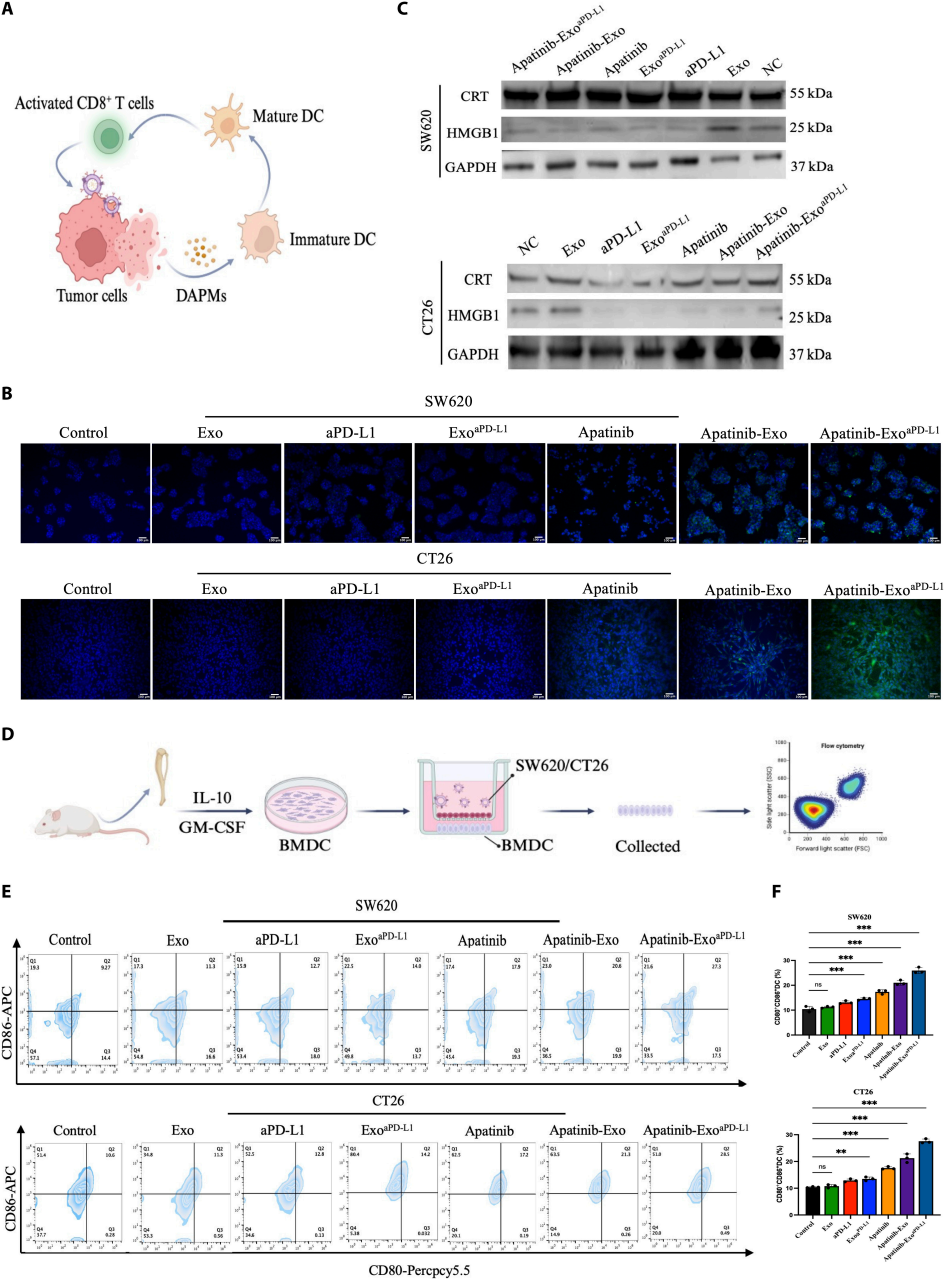
ICD effect induced by Apatinib-Exo^aPD-L1^ and DC maturation in vitro. (A) Schematic illustration of the ICD effect. (B) Fluorescence images of CRT expression in SW620 and CT26 cells after different treatments. (C) Protein expression levels of ICD markers of CRT and HMGB1. (D) Establishment of cell coculture system. (E) Representative flow cytometry analysis indicating DC maturation after different treatments in SW620 and CT26 cells. (F) Quantitative analysis of DC maturation rates.

Apatinib, a selective VEGFR-2 inhibitor, plays a multifaceted role in remodeling the TME to enhance its therapeutic efficacy in cancer treatment [32]. Recent studies have demonstrated that Apatinib modulates the immune microenvironment by reducing the immunosuppressive cells, including Tregs and myeloid-derived suppressor cells (MDSCs), while enhancing the activation and recruitment of cytotoxic T cells [33–35]. However, its potential role to trigger ICD effect remains to be elucidated. To further investigate the ICD effect mediated by Apatinib-Exo^aPD-L1^, we evaluated the surface exposure of CRT and the release of HMGB1, hallmarks of ICD. SW620 and CT26 cells treated with Apatinib-Exo^aPD-L1^ displayed much higher CRT expression and HMGB1 release than the other groups, presumably due to the improved intracellular delivery of Apatinib via exosomal encapsulation (Fig. 3A to C). To further investigate the ICD-mediated DC maturation, SW620 and CT26 cells pretreated with different formulations were cocultured with BMDCs and the expressions of CD80 and CD86 were analyzed by flow cytometry (Fig. 3D). While Apatinib alone moderately promoted DC maturation, Apatinib-Exo^aPD-L1^ induced the highest levels of CD80^+^CD86^+^ DCs, which reached 27.3% and 28.5% in SW620 and CT26 cells, respectively (Fig. 3E and F). These results indicate that Apatinib-Exo^aPD-L1^ enhanced tumor cell immunogenicity and facilitate DC maturation. Taken together, our findings demonstrate that Apatinib-Exo^aPD-L1^ effectively induces ICD in vitro, as evidenced by increased CRT exposure, HMGB1 release, and enhanced DC activation. Since ICD plays a crucial role in converting immunologically “cold” tumors into “hot” ones by releasing DAMPs and TAAs, Apatinib-Exo^aPD-L1^ shows great promise for reprogramming the TME and strengthening adaptive immune responses.

### Apatinib-Exo^aPD-L1^ biodistribution and antitumor efficacy in vivo

**Fig. 4.**
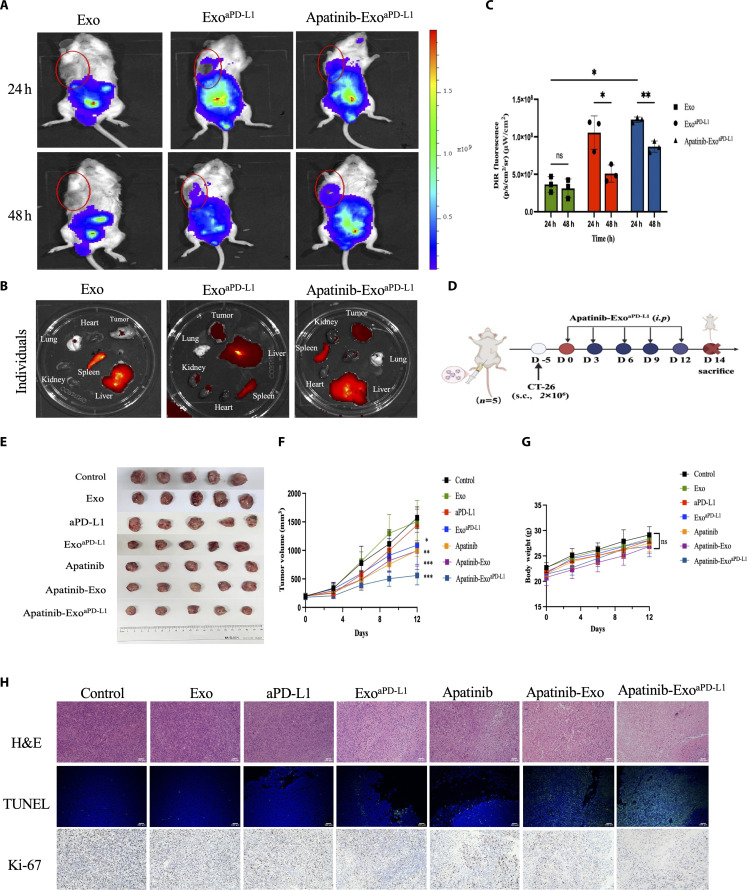
Biodistribution and antitumor efficacy in vivo. (A) In vivo fluorescence images of CT26 tumor-bearing mice at different times after injection of Exo, Exo^aPD-L1^, and Apatinib-Exo^aPD-L1^ (B) Ex vivo fluorescence images of the dissected main organs and tumors at 48 h post-injection. (C) Quantitative analysis of fluorescence efficiency. (D) Systemic illustration of treatment steps in CT26 tumor-bearing mice models. (E) Photographs of excised tumors in different treatment groups on day 19. (F) Body weight curves of mice in each group during different treatments. (G) Tumor growth curves of mice in each group during different treatments. (H) Representative images of H&E, Ki-67, and TUNEL staining of tumor sections after different treatments.

Given the desirable therapeutic efficacy of Apatinib-Exo^aPD-L1^ in vitro, its antitumor performance was further evaluated in vivo. First, to evaluate tumor-targeting efficiency after aPD-L1 modification, DiR-labeled Exo, Exo^aPD-L1^, and Apatinib-Exo^aPD-L1^ were intraperitoneally administered in CT26 tumor-bearing mice. Mice treated with Apatinib-Exo^aPD-L1^ exhibited stronger fluorescence signals at the tumor site compared to those receiving Exo alone. The fluorescence signal peaked at 24 h and gradually decreased thereafter, indicating enhanced tumor targeting and accumulation of Apatinib-Exo^aPD-L1^ in vivo (Fig. 4A and C). Ex vivo imaging of excised tumors and major organs further confirmed that, in addition to accumulation in the liver and kidneys, Apatinib-Exo^aPD-L1^ mainly localized at the tumor sites, demonstrating its superior tumor-targeting specificity as well as prolonged body circulation and retention (Fig. 4B).

Next, the antitumor efficacy of Apatinib-Exo^aPD-L1^ in vivo was evaluated in CT26 tumor-bearing mice according to the treatment protocol illustrated in Fig. 4D. Tumor volume and body weight were monitored throughout the treatment period to assess therapeutic efficacy and biosafety. Compared with the NC group, both Apatinib and Apatinib-Exo induced moderate tumor growth inhibition, whereas Apatinib-Exo^aPD-L1^ exhibited an evident antitumor effect without noticeable systemic toxicity (Fig. 4E to G). Moreover, histological analyses of tumor tissues, including H&E staining, TUNEL assay, and IHC staining (Ki-67), further validated the therapeutic outcomes. H&E staining revealed marked nuclear shrinkage, extensive tumor cell death, and disrupted cellular structure following Apatinib-Exo^aPD-L1^ treatment. Consistently, TUNEL assay and Ki-67 staining demonstrated the highest levels of apoptosis and reduced tumor cell proliferation in the Apatinib-Exo^aPD-L1^ group, confirming its potent antitumor activity in vivo (Fig. 4H). Together, these findings demonstrate that Apatinib-Exo^aPD-L1^ effectively suppresses tumor progression and induces tumor cell apoptosis without systemic toxicity.

### Apatinib-Exo^aPD-L1^ reshapes TME in vivo

**Fig. 5.**
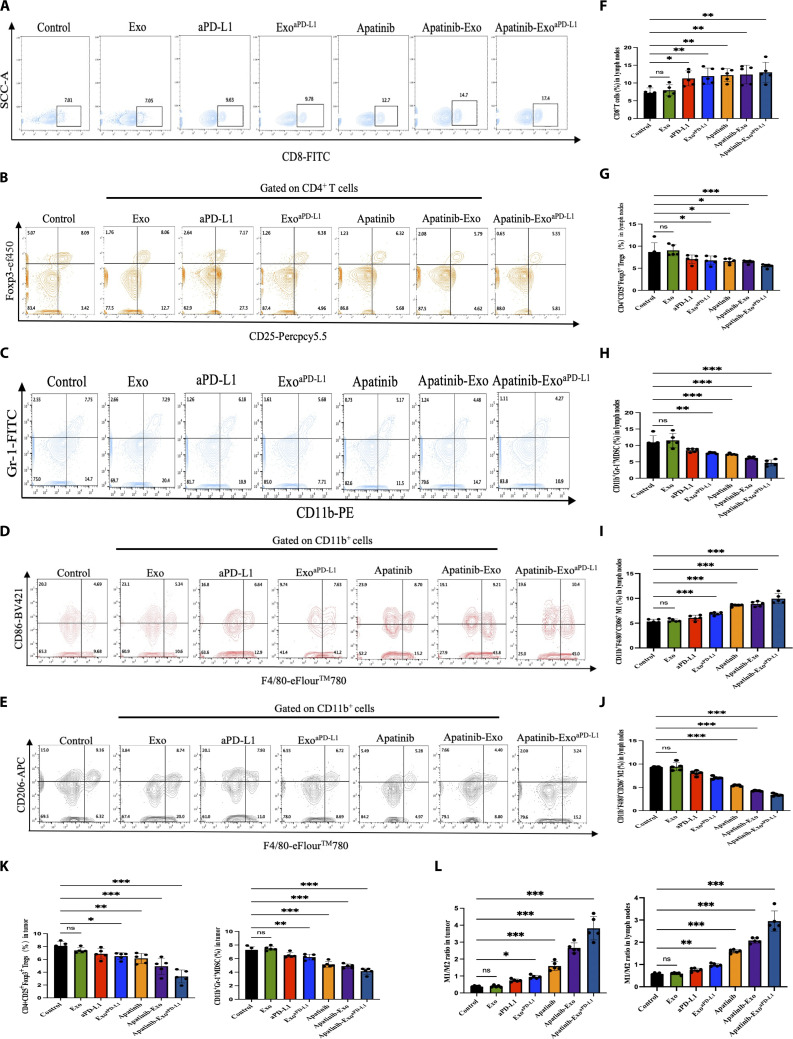
Apatinib-Exo^aPD-L1^ reshapes TME in vivo. (A) Changes of CD8^+^ T cells after different treatments in lymph nodes. (B) Changes of Tregs after different treatments in lymph nodes. (C) Changes of MDSC after different treatments in lymph nodes. (D) Changes of M1 after different treatments in primary tumors. (E) Changes of M2 after different treatments in primary tumors. (F to H) Quantifications of CD8^+^ T cells, Tregs, and MDSCs after different treatments in lymph nodes. (I and J) Quantifications of M1 and M2 after different treatments in tumor tissues. (K) Quantifications of Tregs and MDSCs in tumor tissues. (L) Quantifications of the ratio of M1/M2 in lymph nodes and primary tumors.

**Fig. 6.**
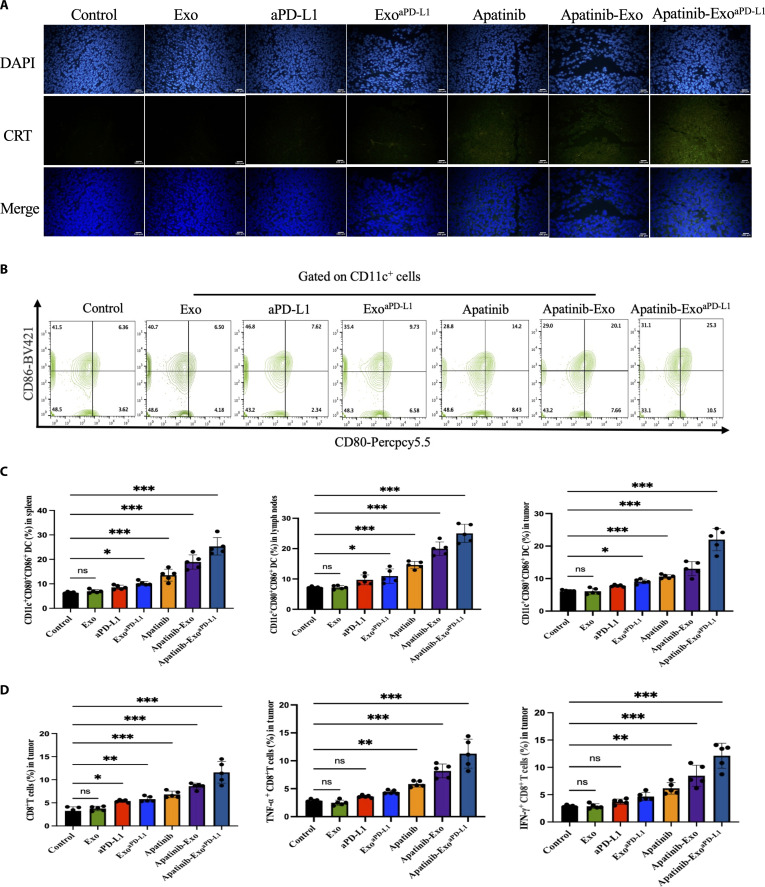
Immune responses mediated by Apatinib-Exo^aPD-L1^. (A) CRT immunofluorescence staining of CT26 tumors excised after 19 d in different treatment groups. (B) Flow cytometry plots of DC extracted from tumor tissue. (C) Changes of DC after different treatments in spleens, lymph nodes, and primary tumors. (D) Proportion changes of TNF-α and IFN-γ in primary tumor tissues.

To elucidate the immune mechanisms underlying the therapeutic effect of Apatinib-Exo^aPD-L1^, the proportions of various immune cells in lymph nodes and tumor tissues were assessed via flow cytometry. Notably, the percentage of CD8^+^ T cells remarkably increased in lymph nodes (11.4%) and tumor tissues (17.4%) in the Apatinib-Exo^aPD-L1^ group, representing 3.29- and 2.48-fold higher than those in the control group, respectively (Figs. 5A and F and 6D). Given that Apatinib has been shown to modulate immunosuppressive cells within the TME [36,37], we further investigated the impact of Apatinib-Exo^aPD-L1^ on immunosuppressive TME. Apatinib-Exo^aPD-L1^ significantly reduced the proportions of CD11b^+^Gr-1^+^ MDSCs and CD4^+^CD25^+^Foxp3^+^ Tregs in lymph nodes by approximately 2-fold and 1.8-fold, respectively, compared to the control group (Fig. 5B, C, G, and H), with similar trends observed in tumor tissues (Fig. 5K). In addition, the ability of Apatinib-Exo^aPD-L1^ to repolarize tumor-associated macrophages (TAMs) from M2-like phenotype toward M1-like phenotype was evaluated in vivo. As expected, Apatinib-Exo^aPD-L1^ remarkably increased the proportion of CD11b^+^F4/80^+^CD86^+^ M1 in tumor tissues, exhibiting 2.21-, 1.36-, and 1.19-fold elevations compared to the control, Exo^aPD-L1^, and Apatinib group, respectively (Fig. 5D and I). In contrast, the proportion of CD11b^+^F4/80^+^CD206^+^ M2 decreased markedly, from 9.16% in the control group to 3.24% in the Apatinib-Exo^aPD-L1^ group (Fig. 5E and J). To our surprise, the M1/M2 ratio increased by approximately 4.9-fold in lymph nodes and 11.7-fold in tumor tissues relative to the control group, significantly surpassing all the other treatment groups (Fig. 5L). Taken together, these results highlight that Apatinib-Exo^aPD-L1^ effectively enhances CTL infiltration and repolarizes TAMs from M2 to M1 phenotype while simultaneously reducing the populations of Tregs and MDSCs in both lymph nodes and tumor tissues. These findings imply the potent immunomodulatory capacity of Apatinib-Exo^aPD-L1^ in reshaping the TME. Nevertheless, further investigation is needed to evaluate its long-term effects and potential immune-related adverse effects.

### Immune responses mediated by Apatinib-Exo^aPD-L1^

To further elucidate the mechanism underlying the immunomodulatory effects of Apatinib-Exo^aPD-L1^ in vivo, the expression of CRT, along with DC maturation in the spleen, lymph nodes, and tumor tissues, was assessed. As shown by immunofluorescence and Western blot analyses, Apatinib-Exo^aPD-L1^ induced the highest level of CRT exposure among all groups, indicating enhanced induction of ICD effect by Apatinib-Exo^aPD-L1^, which may facilitate subsequent DC maturation and antigen presentation, contributing to the robust antitumor immune response (Fig. 6A). Given that DAMPs and TAAs enhance the antigen-presenting capacity of DCs and promote their maturation, we next examined the proportion of mature DCs (CD11c^+^CD80^+^CD86^+^) in spleen, lymph nodes, and tumor sites by flow cytometry. The results showed that the Apatinib-Exo^aPD-L1^ group exhibited the highest maturity rate of DCs, reaching 25.3%, 24.7%, and 20.4%. These results indicate that Apatinib-Exo^aPD-L1^ enhances the tumor antigen presentation and drives robust DC maturation, thereby initiating the strongest immune response in vivo (Fig. 6B and C). As mature DCs are essential mediators linking tumor antigens to CTL activation and infiltration, we further evaluated cytokine secreted by CD8^+^ T cells within the TME. Notably, Apatinib-Exo^aPD-L1^ induced cytokine production of TNF-α and IFN-γ in tumor sites, reaching approximately 3-fold higher than those observed in the control group, suggesting potent immune activation (Fig. 6D).

### Biosafety of Apatinib-Exo^aPD-L1^ in vivo

**Fig. 7.**
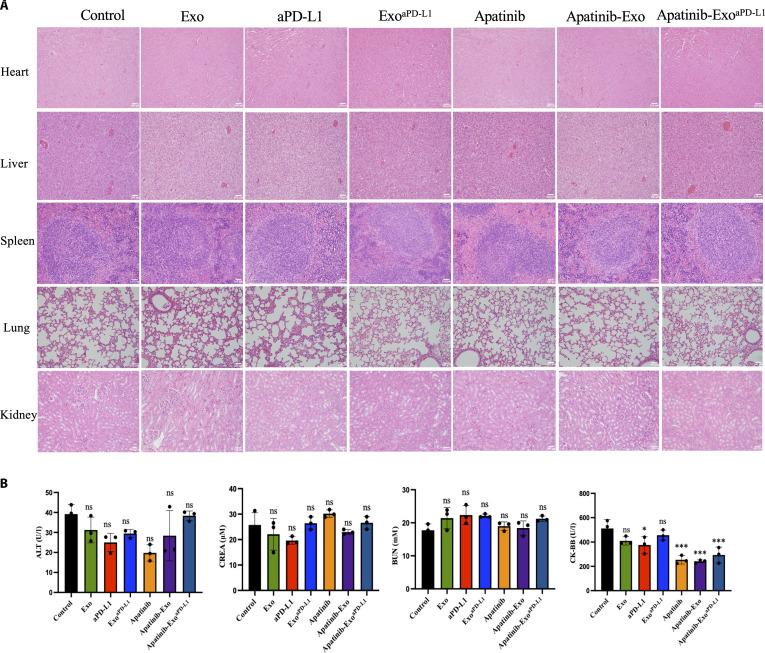
Biosafety of Apatinib-Exo^aPD-L1^ in vivo. (A) H&E staining of the main organs. (B) Biochemical analysis of the serum of mice with different treatments.

Despite favorable therapeutic efficacy achieved by Apatinib-Exo^aPD-L1^, its biosafety in vivo remains an important concern. To evaluate its potential toxicity, the major organs from treated mice were harvested on day 19 and subjected to histopathological examination using H&E staining, along with serum biochemistry analysis. No pathological changes were observed in the heart, liver, spleen, lung, and kidneys across all treatment groups (Fig. 7A). Furthermore, serum biochemistry markers such as alaninea minotransferase (ALT), creatinine (CREA), blood urea nitrogen (BUN), and creatine kinase B (CK-BB) indicated no significant signs of hepatotoxicity, nephrotoxicity, and cardiac toxicity, suggesting that Apatinib-Exo^aPD-L1^ exhibited excellent systemic biocompatibility (Fig. 7B). Notably, a continuous decrease in CK-BB was observed in Apatinib, Apatinib-Exo, and Apatinib-Exo^aPD-L1^ groups compared to the control group, and the underlying reasons for this phenomenon needed to be explored in future studies.

While these findings supported the promising therapeutic potential and systemic biocompatibility of the engineered nanoplatform, several challenges remain to be addressed for future clinical translation. First, the risk of immune-related adverse events, especially those associated with PD-L1 targeted strategies, should be further investigated, and the dose of individual therapeutics needs to be better optimized. The intrinsic biological heterogeneity of exosomes remains a significant barrier for large-scale production. Furthermore, long-term storage stability of exosomes and compliant preparation of antibody-modified exosomes require further investigation. Addressing these limitations will be critical for advancing Apatinib-Exo^aPD-L1^ toward clinical application in cancer therapy.

## Conclusion

**Fig. 8.**
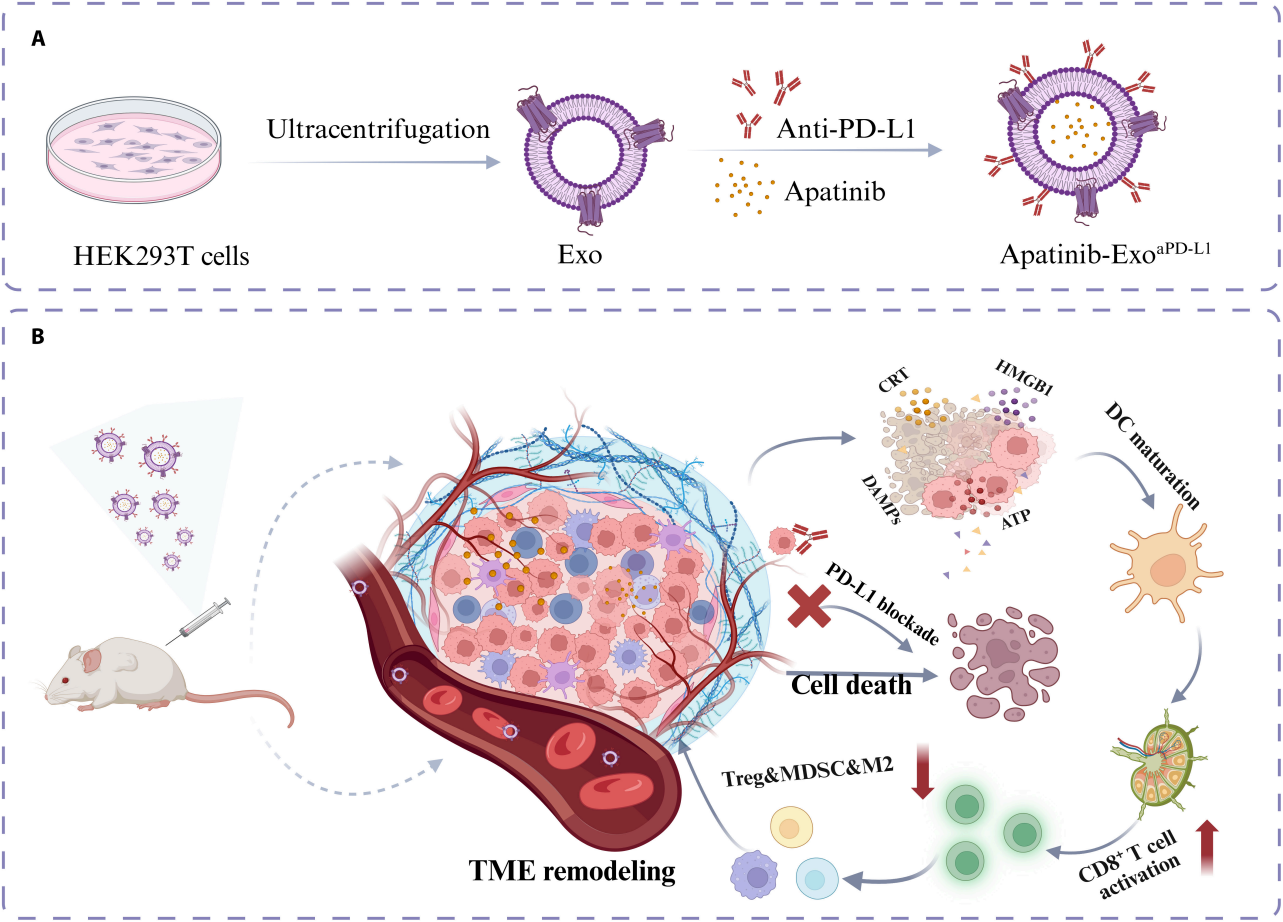
Schematic illustration of Apatinib-Exo^aPD-L1^ boosting antitumor effect and its immune mechanisms against CRC. (A) Schematic illustration of the preparation of Apatinib-Exo^aPD-L1^. (B) Schematic illustration of the antitumor and immunomodulatory mechanisms of Apatinib-Exo^aPD-L1^ in vivo.

Taken together, we successfully developed and characterized a bioengineered exosome-based delivery nanoplatform, Apatinib-Exo^aPD-L1^, for the treatment of CRC (Fig. 8). By integrating aPD-L1 antibody-mediated tumor targeting with the immunomodulatory effects of Apatinib, this nanoplatform exhibited favorable biodistribution, prolonged systemic circulation, and enhanced tumor accumulation. Comprehensive in vitro and in vivo studies confirmed its potent antitumor efficacy, which was attributed to the effective induction of ICD, activation of antitumor immune responses, and suppression of immunosuppressive cells within the TME. Furthermore, the treatment demonstrated excellent biocompatibility and minimal systemic toxicity, highlighting its safety and clinical translational potential. Collectively, these findings provide strong evidence that Apatinib-Exo^aPD-L1^ represents a promising therapeutic strategy for remodeling the immunosuppressive TME of “cold” tumors and improving clinical outcomes in CRC.

## Ethical Approval

The submission reported data involving animal studies were conducted according to the institutional and national guidelines, and the study was approved by the Laboratory Animal Center of the Ningxia Medical University with the approval number IACUC-2024048.

## Data Availability

The datasets supporting the conclusion of this study are available from the corresponding authors upon reasonable request.
