# Supplementary material for: Bioengineered Anti-PD-L1 Functionalized Nanoplatform for Targeted Delivery and Tumor Immune Reprogramming Against Colorectal Cancer
Source: Biomater Res. 2025 Dec 12;29:0284. doi: 10.34133/bmr.0284 (PMC12699071; doi:10.34133/bmr.0284)
Supplement: Supplementary 1 — Table S1 Fig. S1 [file bmr.0284.f1.docx]

**Bioengineered Anti-PD-L1 Functionalized Nanoplatform For Targeted Delivery and Tumor Immune Reprogramming Against Colorectal Cancer**

Miao Liu^1,2†^, Xinjuan Ma^1,2†^, Ruijie Zhou^1†^, Xiaojuan Yang^1^, Yongsheng Zhou^5^, Bin Ma^4*^

Chunxia Su^3*^, Xiangguo Duan^1,2*^

^1^School of Inspection, Ningxia Medical University, Yinchuan, 750004, China;

^2^The First School of Clinical Medicine, Ningxia Medical University, Yinchuan, 750004, China;

^3^School of Basic Medical Sciences, Ningxia Medical University, Yinchuan, 750004, China;

^4^Department of Oncology Surgery, The First People's Hospital of Yinchuan, 750004, China;

^5^The Second School of Clinical Medicine, Ningxia Medical University, Yinchuan, 750004, China;

**Supplementary Tables and Figures:**

**Table S1. Bounding rate of aPD-L1 at various Exo/aPD-L1 ratios**

**
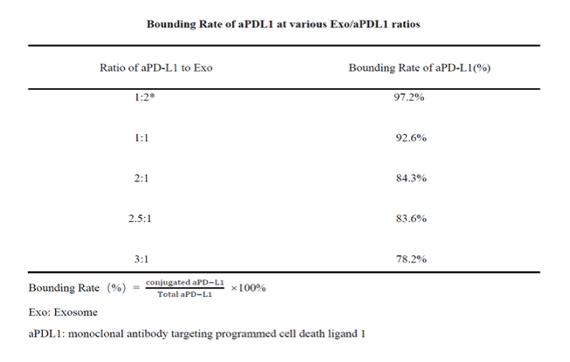
**


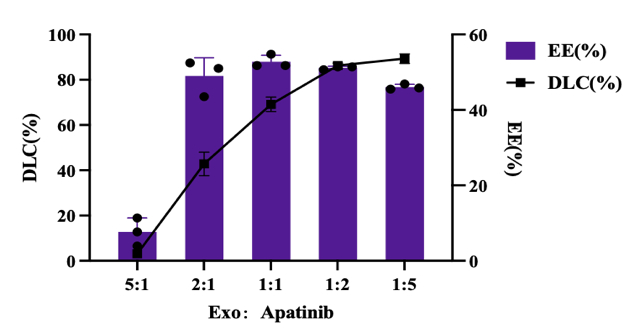
Figure S1.

Figure S1. Quantification of encapsulation efficiency and drug loading capacity of Apatinib
